# Fecal Carriage of *Escherichia coli* Harboring the *tet*(X4)-IncX1 Plasmid from a Tertiary Class-A Hospital in Beijing, China

**DOI:** 10.3390/antibiotics11081068

**Published:** 2022-08-06

**Authors:** Weishuai Zhai, Yingxin Tian, Dongyan Shao, Muchen Zhang, Jiyun Li, Huangwei Song, Chengtao Sun, Yang Wang, Dejun Liu, Ying Zhang

**Affiliations:** 1 Key Laboratory of Animal Antimicrobial Resistance Surveillance, Ministry of Agriculture and Rural Affairs, and Beijing Key Laboratory of Detection Technology for Animal-Derived Food Safety, College of Veterinary Medicine, China Agricultural University, Beijing 100193, China; 2 Department of Laboratory Medicine, the First Medical Centre, Chinese PLA General Hospital, Beijing 100853, China

**Keywords:** tigecycline resistance, *tet*(X4), *Escherichia coli*, IncX1, clonal spread

## Abstract

The emergence of the mobile tigecycline-resistance gene, *tet*(X4), poses a significant threat to public health. To investigate the prevalence and genetic characteristics of the *tet*(X4)-positive *Escherichia coli* in humans, 1101 human stool samples were collected from a tertiary class-A hospital in Beijing, China, in 2019. Eight *E. coli* isolates that were positive for *tet*(X4) were identified from clinical departments of oncology (*n* = 3), hepatology (*n* = 2), nephrology (*n* = 1), urology (*n* = 1), and general surgery (*n* = 1). They exhibited resistance to multiple antibiotics, including tigecycline, but remained susceptible to meropenem and polymyxin B. A phylogenetic analysis revealed that the clonal spread of four *tet*(X4)-positive *E. coli* from different periods of time or departments existed in this hospital, and three isolates were phylogenetically close to the *tet*(X4)-positive *E. coli* from animals and the environment. All *tet*(X4)-positive *E. coli* isolates contained the IncX1-plasmid replicon. Three isolates successfully transferred their tigecycline resistance to the recipient strain, C600, demonstrating that the plasmid-mediated horizontal gene transfer constitutes another critical mechanism for transmitting *tet*(X4). Notably, all *tet*(X4)-bearing plasmids identified in this study had a high similarity to several plasmids recovered from animal-derived strains. Our findings revealed the importance of both the clonal spread and horizontal gene transfer in the spread of *tet*(X4) within human clinics and between different sources.

## 1. Introduction

In recent decades, antimicrobial resistance (AMR) in clinical pathogens has become a significant threat to human health and a major source of concern for microbiologists and clinicians around the world. Tigecycline, the first antibiotic of the glycylcycline class, is considered one of the last antibiotic options for treating clinical infections caused by multi-drug resistance (MDR) Gram-negative bacteria, particularly carbapenem-resistant Enterobacteriaceae (CRE) and carbapenem-resistant *Acinetobacter baumannii* (CRAB) [1]. However, the frequent use of tigecycline promotes the development of tigecycline resistance, which can lead to a clinical treatment failure. Earlier studies found that tigecycline resistance is typically generated by the over-expression of efflux pumps and mutations within the drug-binding site in the ribosome [2], but there have been few reports of a horizontal gene transfer of tigecycline resistance. 

The plasmid-carried *tet*(A) mutations can lead to a low-level resistance to tigecycline in *Klebsiella pneumoniae* [3], but it is uncommon. Tet(X), a flavin-dependent monooxygenase that can inactivate tetracyclines, was first described in *Bacteroides fragilis* [4,5]. Tet(X2), an ortholog of Tet(X), was originally isolated from the transposon CTnDOT in *Bacteroides thetaoiotaomicron* [6]. Both *tet*(X) variants are active against the earlier classes of tetracyclines but show limited activity against tigecycline [7]. In 2019, two novel plasmid-encoded mobile tigecycline-resistance genes, *tet*(X3) and *tet*(X4), were initially discovered in *A. baumannii* and Enterobacteriaceae isolated from animals, humans, and the environment in multiple provinces of China [8,9]. These two variants confer a high-level resistance to all tetracycline antibiotics, including tigecycline as well as two FDA-approved new antibiotics, eravacycline and omadacycline [8]. Following that discovery, several novel *tet*(X) variants, *tet*(X5) [10], *tet*(X6) [11], *tet*(X7) to *tet*(X13) [12], *tet*(X14) [13], and *tet*(X15) [14], have been identified in a variety of bacterial species from diverse sources. 

Fortunately, most *tet*(X) variants have only been reported sporadically and cannot be transferred by a plasmid-mediated horizontal gene transfer. However, it should be noted that the *tet*(X4) gene has gradually become one of the most common plasmid-mediated tigecycline genes in China [15] and has also been identified in more than five countries in Europe and Asia [16,17]. The *tet*(X4) gene was predominantly found in pigs, pork, and the surrounding environments of pig farms or slaughterhouses but was rare in human health sectors. Unfortunately, the presence of *tet*(X4) progressively increased in clinical cases [18,19] and in healthy humans [15]. Moreover, a greater concern is that the *tet*(X4) gene has been sporadically found in coexistence with the mobile colistin gene, *mcr-1,* [20] or carbapenemase-encoding gene *bla*_NDM-5_ [21], further limiting the drug options for the treatment of infections caused by these extensively drug-resistant bacterial pathogens. Notably, the rapid spread of the *tet*(X4) gene between different clinical strains was attributed to horizontal gene transfers within hospitals [18,19], but the clonal transmission of *tet*(X4) between different sources, particularly animals and humans, cannot be neglected even if it is relatively rare.

In this study, we described the antibiotic-resistance characteristics and molecular epidemiology of the clinical *tet*(X4)-positive *E. coli* isolates in a Chinese hospital. We further identified that the *tet*(X4) gene can be transmitted via bacterial clonal spread or horizontal genetic transfer in hospitals. These findings will help us in better understanding the transmission of *tet*(X4) between animals and humans.

## 2. Results

### 2.1. Resistance Genes, Plasmid Replicons, and Virulence Factors

A total of 1101 fresh faeces were collected through a four-month surveillance programme (from June to September) in a tertiary class-A hospital in 2019. Eight tigecycline-resistant isolates that were positive for the *tet*(X4) gene were identified as *Escherichia coli*. They were isolated from the fecal samples of six male and two female hospitalized patients in June (*n* = 1), August (*n* = 5), and September (*n* = 2), respectively. These patients came from five distinct clinical departments: oncology (*n* = 3), hepatology (*n* = 2), nephrology (*n* = 1), urology (*n* = 1), and general surgery (*n* = 1) (Table S1). 

**Figure 1:**
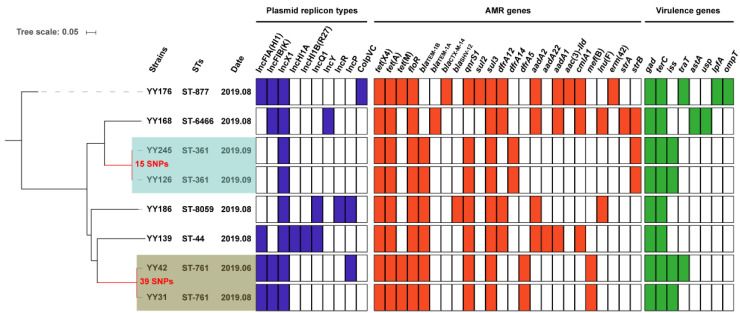
The phylogenetic tree and genomic features of eight *tet*(X4)-positive *E. coli* isolates. On the phylogenetic tree, the light blue and light brown color ranges reflect the two clonal groups. The heatmap in different colors depicts the presence or absence of the plasmid replicon types (blue), antimicrobial-resistance (AMR) genes (red), and virulence genes (green).

The antimicrobial-susceptibility test revealed that eight *tet*(X4)-positive *E. coli* isolates displayed resistance to multiple antibiotics, including ampicillin, doxycycline, tigecycline, sulfamethoxazole-trimethoprim, and florfenicol, while still being susceptible to polymyxin B and meropenem (Table S1). In addition, two *E. coli* isolates (YY176 and YY139) were resistant to levofloxacin and ciprofloxacin, but only one isolate, YY176, was also resistant to gentamicin and ceftriaxone. All of the *tet*(X4)-positive *E. coli* isolates carried the sulphonamide (*sul3*), trimethoprim (*dfrA*), phenicol (*floR*), and tetracycline (*tet*(A) and *tet*(X4)) resistance genes and at least one β-lactamase resistance gene (such as *bla*_TEM-1A_, *bla*_TEM-1B_, *bla*_CTX-M-14_, and *bla*_SHV-12_) (Figure 1), which was basically consistent with the presence of their resistance phenotypes. 

The PlasmidFinder analysis of eight isolates identified ten distinct plasmid replicons: IncFIA(HI1), IncFIB(K), IncX1, IncHI1A, IncHI1B(R27), IncQ1, IncY, IncR, IncP, and ColpVC (Figure 1). IncX1 was identified in each of these isolates. In addition, eight virulence genes, including *gad* (*n* = 8), *terC* (*n* = 8), *iss* (*n* = 4), *traT* (*n* = 2), *astA* (*n* = 1), *usp* (*n* = 1), *lpfA* (*n* = 1), and *ompT* (*n* = 1), were identified. Only one *E. coli* YY176 isolate exhibited five virulence genes, and the remaining seven isolates possessed two to four virulence genes (Figure 1). Although the presence of virulence genes does not indicate pathogenicity, it still implies a pathogenic potential, which poses a potential threat to human health. 

### 2.2. Genomic Population Structure and the Phylogenetic Context

**Figure 2:**
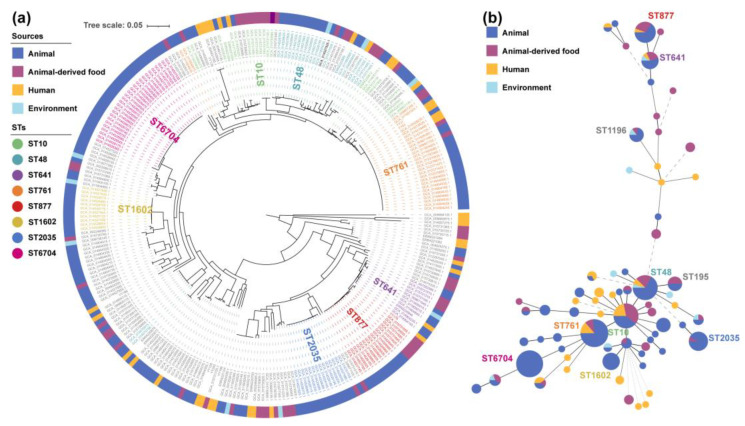
Phylogenetic tree (**a**) and minimum spanning tree (**b**) of 280 *tet*(X4)-positive *E. coli*.

The MLST analysis revealed that eight *tet*(X4)-carrying isolates had a high degree of genetic diversity and could be classified into six sequence types (STs), including ST8059 (*n* = 1), ST6466 (*n* = 1), ST877 (*n* = 1), ST761 (*n* = 2), ST361 (*n* = 2), and ST44 (*n* = 1) (Figure 1 and Figure 2). Two STs (ST877 and ST761) have already been discovered in animal- and human-derived *tet*(X4)-positive *E. coli* [17,18,22]. The SNP analysis revealed that these isolates shared a total of 65,529 single nucleotide polymorphisms (SNPs), with SNPs ranging from 15 to 49,573 bp between them (Table S2). *E. coli* YY126 and YY245 shared the fewest SNPs (15 SNPs), followed by *E. coli* YY31 and YY42 (39 SNPs), indicating that these strains had a closer genetic relationship. Interestingly, *E. coli* YY126 and YY245 were isolated concurrently, but they originated in separate sections (oncology and hepatology) within the hospital. By contrast, *E. coli* YY31 and YY42 were isolated at separate times from distinct clinical departments in the hospital. These findings suggest that a portion of the *tet*(X4)-positive *E. coli* could be spreading clonally within this hospital. 

To further explore the potential origin of the eight clinical isolates, a phylogenetic tree was generated using our eight and two-hundred and seventy-two online *tet*(X4)-positive *E. coli* genomes based on a core-genome SNP analysis (Figure 2a), and a minimum spanning tree was constructed using the MLST data (Figure 2b). There were over 70 different STs among the 280 strains, with 8 dominant STs (more than ten strains) accounting for 55% (95% CI, 49.1–60.9%). However, only three STs (ST761, ST877, and ST641) have great potential in the transmission between humans and animals via clonal spread among the eight dominant STs. Most of them were not only obtained from multiple sources but also have a very close evolutionary distance in the individual groups (Figure 2a), especially the previously proved *E. coli* ST761 [23]. In this study, two clonal groups and four non-clonal *E. coli* isolates were located on six separate clades in the phylogenetic tree. Three out of eight isolates belonging to ST761 or ST877 were phylogenetically closely related to the strains from animals and the environment, while the remaining five isolates clustered alone. Therefore, taken as a whole, the transmission of the *tet*(X4) gene in this hospital may be associated with both the clonal spread and horizontal gene transfer. 

### 2.3. Conjugation and the Genetic Environment

The horizontal transmissibility of the *tet*(X4)-bearing plasmid was determined via a conjugation assay. Three isolates, *E. coli* YY42, YY168, and YY186, successfully transferred their tigecycline resistance to the recipient strain, *E. coli* C600, with transfer frequencies ranging from 2.39×10^−7^ to 1.32 × 10^−4^ (Table S1 and Figure S1), whereas the remaining five strains failed. To better understand the horizontal gene transfer of the *tet*(X4) gene, a third-generation sequencing was performed on *E. coli* YY42, YY168, and YY186 with their transconjugants TCYY42, TCYY168, and TCYY186. However, *E. coli* YY42 and its transconjugant *E. coli* TCYY42 were unable to acquire the entire *tet*(X4)-bearing plasmid sequence due to the multiple tandem repeats of the *tet*(X4)-bearing sequence. Thus, the two genomes were reassembled based on the available long-read data to obtain the complete plasmid sequence. In fact, such tandem repeats are common in the *tet*(X4)-positive strains and frequently result in a failed assembly of the *tet*(X4)-bearing plasmid, as previously described [19,24,25].

We further analysed the genetic context of the *tet*(X4) gene among the eight isolates to observe the possible tandem repeats. The IS*CR2* was detected on the downstream-flanking region of *tet*(X4) in all isolates, but there were several cases of the upstream-flanking region of *tet*(X4) (Figure S2). One such case showed that IS*CR2* was absent or IS*CR2* was terminated by other mobile elements on the upstream-flanking region of *tet*(X4), which does not affect the normal assembly of the *tet*(X4)-bearing plasmid. Alternatively, two entire IS*CR2* were positioned on both the upstream- and downstream-flanking regions of *tet*(X4), which could form numerous tandem repeats and ultimately lead to an assembly failure. In short, the presence or absence of the IS*CR2* upstream of the *tet*(X4) gene is a critical determinant of the assembly of the *tet*(X4)-bearing plasmid. Subsequently, the plasmid analyses of eight isolates using both the hybrid assembly and only-long-read assembly revealed that four *tet*(X4)-bearing IncX1 plasmids, pYY168, pYY186, YY245, and pYY126_trycycler, with sizes ranging from 40 to 60 kb, were identified in four *E. coli* isolates; three *tet*(X4)-bearing IncX1/FIA(HI1)/FIB(K) hybrid plasmids, pYY31, pYY42_trycycler, and pYY176_trycycler, with sizes ranging from 120 to 180 kb, were identified in three *E. coli* isolates; one 248,932 bp *tet*(X4)-bearing IncX1/FIA(HI1)/HI1A/HI1B(R27) hybrid plasmid, pYY139, was identified in one isolate.

### 2.4. The tet(X4)-Carrying IncX1 Plasmid

**Figure 3:**
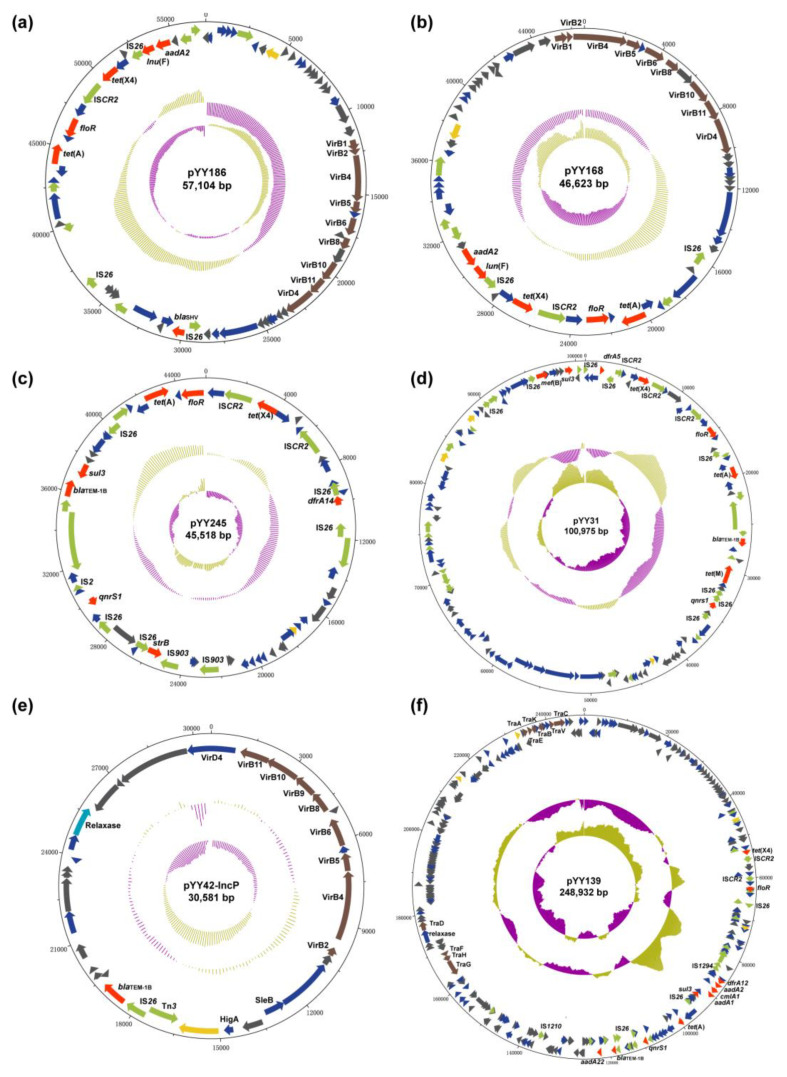
Schematic maps of multiple plasmids. A circular map of pYY186 (**a**), pYY168 (**b**), pYY245 (**c**), pYY31 (**d**), pYY42-IncP (**e**), and pYY139 (**f**). The innermost circle presents the GC-Skew and the middle circle presents the GC content. The gene functions are indicated by arrows with different colors in the outer circle. Red, AMR gene; yellow, replication initiation protein gene; brown, conjugative transfer gene; green, mobile element; dark grey, hypothetical protein gene; navy blue, other functional gene.

**Figure 4:**
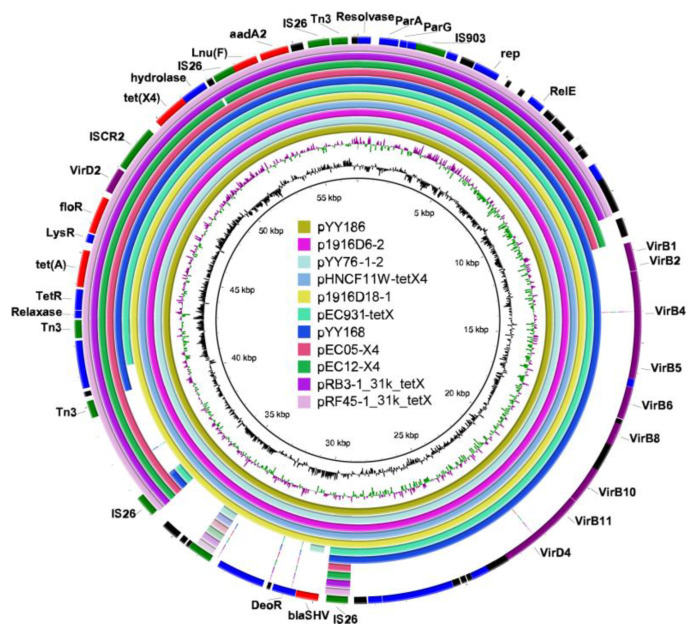
Circular comparison of the *tet*(X4)-bearing IncX1 plasmids with other closely related IncX1 plasmids from the NCBI database. The outermost ring represents the reference IncX1 plasmid pYY186 with its gene positions. Different colors in the outermost ring represent distinct genes: Red represents the resistance gene, purple represents the gene of T4SS, green represents the mobile element, black represents the hypothetical protein, and blue represents other functional genes. The map was constructed using BRIG software.

The analysis of two transferable *tet*(X4)-carrying IncX1 plasmids showed that pYY168 and pYY186 are composed of three components: a plasmid backbone and two variable regions, including one multidrug-resistance determining region and one conjugative-transfer determining region that contained a VirB family type IV secretion system (T4SS) (Figure 3a,b). The multidrug-resistance determining region of pYY186 possessed six distinct AMR genes, including *tet*(X4), *tet*(A), *floR*, *lnu*(F), *aadA2*, and *bla*_SHV-12_, but pYY168 lacked *bla*_SHV-12_. Through a Blastn alignment, we determined that pYY186 shared 99.97% sequence identity with pYY168 at 83% coverage (Figure 4). Four online IncX1 plasmids from the NCBI database were acquired using pYY186 as a reference query (99% identity and 100% coverage): p1916D6-2 (59,351 bp, accession no. CP046002), pYY76-1-2 (57,105 bp, accession no. CP040929), pHNCF11W-tetX4 (57,104 bp, accession no. CP053047), and p1916D18-1 (59,353 bp, accession no. CP045998) (Figure 4). These plasmids were recovered from four *E. coli* strains isolated from cows (*n* = 1), chicken (*n* = 1), and swine (*n* = 2). We also found one *tet*(X4)-bearing plasmid, pEC931_tetX (50,626 bp, accession no. CP049121), from one *E. coli* strain from a person with a urinary tract infection that shared a 97% identity with 99.97% coverage with pYY168 and an 81% identity with 100% coverage with pYY186. Moreover, another four NCBI-obtained *tet*(X4)-bearing IncX1 plasmids (accession no. NZ_MN436006, NZ_MN436007, NZ_MT197111, and NZ_MT219821) ranging from 30 to 40 kb in length shared 57% identity and >99.97% coverage with pYY186, but these plasmids lacked the VirB family T4SS, which may result in the functional absence of conjugation (Figure 4). Notably, in the current study, two *tet*(X4)-bearing IncX1 plasmids, pYY245 and pYY126_trycycler, lacking the conjugation capacity were also devoid of the VirB family T4SS. They were 99.97% identical to two 44,691 bp plasmids, pCD58-3-1(accession no. CP050037) and pCD74-2-2 (accession no. CP050046), recovered from the *E. coli* strains of a broiler chicken, at 100% coverage, and carried eight AMR genes including *tet*(X4), *tet*(A), *bla*_TEM-1B_, *aph*(6)*-ld*, *floR*, *sul3*, *qnrS1*, and *dfrA14* (Figure 3c and Figure S3). The high similarity of the *tet*(X4)-bearing IncX1 plasmid between animals and humans indicated that the plasmid has achieved a wide distribution among different origins and plays an important role in the transmission of multidrug resistance, including tigecycline resistance.

### 2.5. The tet(X4)-Carrying IncX1-containing Hybrid Plasmid

By conducting a Blastn alignment of three IncX1/FIA(HI1)/FIB(K) hybrid plasmids, we established that pYY31 was virtually identical to the long-read assembly plasmid, pYY42_trycycler (>99% identity and 100% coverage) (Figure 3d and Figure S4a), but differed from the long-read assembly plasmid pYY176_trycycler. Using pYY31 as a reference query, more than ten plasmids ranging from 100 to 130 kb recovered from pigs and cattle were retrieved using BLASTn (>99% identity and 100% coverage). These shared a highly similar plasmid backbone and nine AMR genes, including *tet*(X4), *tet*(A), *tet*(M), *bla*_TEM-1B_, *mef*(B), *floR*, *sul3*, *qnrS1*, and *dfrA5*. Interestingly, both pYY42_trycycler and pYY31 lacked the conjugative-elements VirB family T4SS, but pYY42_trycycler could be transformed into the recipient strain by conjugation. A further analysis of the full genome sequences revealed the presence of a 30,581 bp IncP plasmid, pYY42-IncP (Figure 3e and Figure S4b), in *E. coli* YY42 and its transconjugants TCYY42, but not in *E. coli* YY31. The plasmid, pYY42-IncP, contained the VirB family T4SS, which may aid in the co-transfer of the plasmid pYY42_trycycler to recipient strains via conjugation. It was 100% identical to a 30,581 bp plasmid, pD72-IncP (accession no. CP035316.1), recovered from an animal-derived *mcr-1*-positive *E. coli* isolate D72 at 100% coverage (Figure S4b). In addition, the long-read assembly plasmid, pYY176_trycycler, was more than 99% identical to four plasmids recovered from the *E. coli* strains of pigs at >87% coverage (Figure S4c). This plasmid carried 11 AMR genes including *tet*(X4), *tet*(A), *tet*(M), *aadA1*, *aadA2*, *floR*, *erm*(42), *cmlA1*, *sul2*, *sul3*, and *dfrA12*. The IncX1/FIA(HI1)/HI1A/HI1B(R27) hybrid plasmid, pYY139 (Figure 3f), was 100% identical to the 219,101 bp plasmid, p1919D3-1 (accession no. CP046004), recovered from an *E. coli* isolate of swine feces, at 89% coverage (Figure S4d). This plasmid carried a multidrug-resistance region that included *tet*(X4), *tet*(A), *aadA1*, *aadA2*, *aadA22*, *bla*_TEM-1B_, *qnrS1*, *floR*, *cmlA1*, *sul3*, and *dfrA12*. Overall, these results suggested that the VirB family T4SS played an important role in the transmission of the *tet*(X4)-bearing IncX1 plasmid or IncX1-containing hybrid plasmid.

## 3. Discussion

Since the plasmid-mediated tigecycline-resistance gene *tet*(X4) was first described in China in 2019 [8,9], it has been widely detected in animals but sporadically reported in humans. The earliest report revealed that the detection rate of *tet*(X4)-positive *E. coli* isolates in humans was only 0.07% [8] (4/5485). This proportion increased to 0.73% (8/1101) among clinical *tet*(X4)-positive *E. coli* isolates in our study. Likewise, some recent studies found that the proportion of clinical *tet*(X4)-positive *E. coli* in humans increased modestly [15,18], but it was unclear whether the increase was associated with the high prevalence of *tet*(X4) in animals. Therefore, monitoring their reservoirs and transmission routes is essential, particularly regarding cross-species transmission between animals and humans.

Current epidemiological evidence indicated that the *tet*(X4) gene was predominantly presented in Enterobacteriaceae, particularly *E. coli* [8,9,15,17,18,26]. Thus, using *E. coli* as a model species for investigating the transmission of tigecycline resistance between diverse sources is preferred. According to a previous study, twelve *tet*(X4)-positive *E. coli* isolated from the gut microbiota of healthy Singaporeans possessed nine known STs and three untypable STs [17]. In addition, clinical isolates of *tet*(X4)-positive *E. coli* also exhibited a significant degree of genetic diversity in certain areas of China [18]. Hence, the rapid acquisition and dissemination of *tet*(X4) are commonly attributed to a horizontal gene transfer via conjugative plasmids and the translocation of the active mobile element, IS*CR2* [26,27], rather than to clonal spread. Recently, several dominant clonal types of *E. coli* (such as *E. coli* ST10 and ST48) carrying *tet*(X4) have been detected in both animals and humans [18,22]; however, the SNP numbers often differ widely between different sources according to the SNP analysis of this study. Notably, unlike these STs, two *E. coli* ST761 isolates in this study shared a close relationship with other *tet*(X4)-positive *E. coli* ST761 strains isolated from animal-derived samples [23]. We also discovered that one isolate shared a significant degree of genetic similarity with a *tet*(X4)-positive *E. coli* ST877 strain isolated from pork. These findings suggest that the clonal spread of *tet*(X4)-positive dominant clonal types across humans and animals poses a great threat to human health. 

The IncX-group plasmids, especially IncX3 [28] and IncX4 [29], demonstrated an important role in contributing to the spread of carbapenemase genes and colistin-resistance genes between different strains. Currently, more than eight plasmid-replicon types were observed in the *tet*(X4)-positive *E. coli* strains [22,24,26]. Of these, IncX1 plasmids have been found in diverse STs of *E. coli* from multiple sources [8,9,15,18,22,24]. All of the *tet*(X4)-bearing plasmids in this study were IncX1 plasmids or IncX1-containing hybrid plasmids; both groups had a high similarity to several plasmids recovered from animal-derived strains, suggesting a connection of these plasmids between the strains from humans and animals. Notably, the IncX1 plasmid was able to form a hybrid plasmid with other Inc plasmids (e.g., IncF plasmid), which facilitated its survival in a broad range of hosts. Although some IncX1 plasmids have lost the capacity for self-conjugation, their conjugation can occur through a helper plasmid carrying a VirB family T4SS, such as IncP, as observed in this study. Therefore, we propose that IncX1 plasmids or IncX1-containing hybrid plasmids play a significant role in the dissemination of the *tet*(X4) gene.

## 4. Materials and Methods

### 4.1. Sample Collection and Strain Identification

Fresh fecal samples were collected from patients for antibiotic-resistance surveillance at a tertiary class-A hospital in Beijing, China, from June to September 2019 (Table S3). The fresh samples were homogenized in PBS (pH = 7.2). The 100 μL sample of homogenate was then mixed with 10 mL of LB broth (supplemented with 2 mg/L tigecycline) and incubated for 12 h at 37 °C with 200 rpm shaking. Next, the enriched broth was streaked on CHROMagar^TM^ Orientation agar plates with tigecycline (2 mg/L) and incubated at 37 °C for 24 h. Purified colonies were obtained after re-streaking three times on a MacConkey agar plate and were then stored in a Microbank^TM^ (Pro-Lab Diagnostics, Toronto, ON, Canada) at −70 °C. Bacterial species were identified using a MALDI-TOF/MS (Shimadzu, Kyoto, Japan) and reconfirmed by 16S rRNA gene sequencing. A colony PCR was used to screen *tet*(X)-positive clones using the universal primers, *tet*(X)-F (5′-TGA ACC TGG TAA GAA GAA GTG-3′) and *tet*(X)-R (5′-CAG ACA ATA TCA AAG CAT CCA-3′), and Sanger sequencing was used to confirm all amplicons after PCR amplification.

### 4.2. Antimicrobial Susceptibility Testing

The minimum inhibitory concentrations (MICs) were determined using the broth microdilution method following the latest guidelines of the Clinical and Laboratory Standards Institute (CLSI) and the European Committee on Antimicrobial Susceptibility Testing (EUCAST). Briefly, all isolates were first streaked onto MHA agar and grown overnight at 37 °C. Then, each isolate was inoculated in 0.9% NaCl to a McFarland standard of 0.5 and was tested for susceptibility to 12 antibiotics using custom-made Sensititre plates (Thermo Fisher Scientific, USA), including ampicillin (AMP), amoxicillin-clavulanate (2:1) (AMC), doxycycline (DOX), tigecycline (TGC), levofloxacin (LVX), ciprofloxacin (CIP), ceftriaxone (CRO), gentamicin (GEN), meropenem (MEM), trimethoprim-sulfamethoxazole (SXT), florfenicol (FFC), and polymyxin B (PB). Finally, all isolates were incubated for 16–20 h at 37 °C. The resistance breakpoints of most antimicrobial drugs were interpreted according to the CLSI guidelines [30], whereas tigecycline MIC was defined by the EUCAST breakpoints [31] for *E. coli*. ATCC25922 was used as a reference strain (quality control).

### 4.3. Conjugation Assay

The transmission of the *tet*(X4) gene was assessed by performing the conjugation experiment using the filter-mating method with the streptomycin-resistant *E. coli* C600 as the recipient. Briefly, donor and recipient strains were grown overnight and then diluted at 1:100 in fresh LB broth. After a 6 h incubation at 37 °C, the donor and recipient strains were mixed at a 1:3 ratio. The mixtures were subsequently coated on a 0.45 μM microporous composite membrane on a solid medium and incubated at 37 °C for 6 h. Transconjugants were selected on MacConkey agar plates containing 2 mg/L tigecycline with 3000 mg/L of streptomycin and were verified by PCR to confirm the successful transfer. Transfer frequencies were calculated as the number of transconjugants obtained per recipient, as previously described [8].

### 4.4. Genome Sequencing and Bioinformatics

The whole-genome DNA of all isolates and transconjugants were extracted using a HiPure Bacterial DNA Kit (Magen, Guangzhou, China) following the protocols described by the manufacturer. Samples were sent to Sinobiocore (Beijing, China) for sequencing on the Illumina HiSeq 2500 system with a read length of 150 bp, paired-end. Then, Nanopore libraries were constructed and sequenced on the MinION long-read sequencing platform (Oxford Nanopore Technologies, Oxford, UK). Both Illumina short reads and Oxford Nanopore long reads of each strain were included in a hybrid assembly using a Unicycler (Version 4.0.1, https://github.com/rrwick/Unicycler, accessed on 1 July 2022) [32]. Three plasmids were reassembled using a Trycycler (Version 0.5.3, https://github.com/rrwick/Trycycler, accessed on 1 July 2022) due to the failed assembly of the *tet*(X4)-bearing plasmids caused by multiple tandem repeats [33]. After assembling, medaka (Version 1.4.3, https://github.com/nanoporetech/medaka, accessed on 1 July 2022) and pilon (Version 1.2.4, https://github.com/broadinstitute/pilon, accessed on 1 July 2022) [34] were used to polish the plasmid sequences. Online genomes of *tet*(X4)-carrying *E. coli* were obtained from the NCBI database (Table S4). The AMR determinants, plasmid replicons, and sequence types were identified using a Staramr (Version 0.5.1, https://github.com/phac-nml/staramr, accessed on 1 July 2022) [35] against the ResFinder [36], PlasmidFinder, and MLST databases [37], respectively. Gene prediction and automatic annotation were performed using the RAST service [38]. Putative virulence determinants were identified using VirulenceFinder (version 2.0, https://cge.food.dtu.dk/services/VirulenceFinder, accessed on 1 July 2022) [39]. A minimum spanning tree of all sequence types was constructed in the BioNumerics software (version 7.0, https://www.applied-maths.com/bionumerics, accessed on 1 July 2022) according to correlations among alleles. Phylogenetic trees were performed using the Parsnp (Harvest v1.1.2, https://github.com/marbl/parsnp, accessed on 1 July 2022) and visualized using iTOL (https://itol.embl.de, accessed on 1 July 2022). Plasmid maps were manually annotated using the DNAplotter software [40] and the comparison analysis of multiplex plasmid sequences was performed using a BLAST Ring Image Generator (BRIG, http://brig.sourceforge.net/, accessed on 1 July 2022) [41].

### 4.5. Statistical Analysis

Data were collected using Microsoft Excel files. A statistical analysis was performed with the IBM SPSS Software, version 25 (IBM SPSS Statistics, Armonk, NY, USA). The confidence interval (CI) reported was at 95%.

## 5. Conclusions

In conclusion, we characterized the epidemiological and genomic features of *tet*(X4)-positive *E. coli* isolated from the stool of inpatients from a tertiary class-A hospital in China. The clonal spread of the *tet*(X4)-positive isolates indicated the risk of intra-hospital transmission of the *tet*(X4) gene. In addition, although specific origins could not be accurately traced, these strains and plasmids of clinic patient origin showed a strong genetic resemblance to some animal-origin strains, implying a potential risk of transmission between animals and humans. As such, since both the clonal spread and horizontal gene transfer aggravate the spread of the *tet*(X4) gene, the routine surveillance of the *tet*(X) genes is critical for effectively curbing the further transmission of tigecycline-resistance strains between animals and humans.

## Data Availability

All genome sequences have been deposited in the GenBank database under the BioProject accession number, PRJNA846553. The sequence data of all transconjugants and three only-long-read assembled plasmids were deposited in the figshare database (https://doi.org/10.6084/m9.figshare.20071841, accessed on 1 July 2022) for reference.

## Supplementary Materials

The following supporting information can be downloaded at: https://www.mdpi.com/article/10.3390/antibiotics11081068/s1, Figure S1: Distribution of antimicrobial-resistance genes and plasmid-replicon types among three *E. coli* isolates and their transconjugants; Figure S2: Characterization of *tet*(X4)-bearing genetic environments in eight isolates; Figure S3: Circular comparison of *tet*(X4)-bearing IncX1 plasmid, pYY245; Figure S4: Circular comparison of four plasmids. Table S1: MICs of eight *tet*(X4)-positive isolates and three transconjugants; Table S2: Pairwise SNP distance matrix for eight *tet*(X4)-positive isolates; Table S3 Information of 1101 fecal samples; Table S4: Information of eight *tet*(X4)-positive *E. coli* and two-hundred and seventy-two online *tet*(X4)-positive *E. coli* genomes.
