# Supplementary material for: Fecal Carriage of Escherichia coli Harboring the tet(X4)-IncX1 Plasmid from a Tertiary Class-A Hospital in Beijing, China
Source: Antibiotics (Basel). 2022 Aug 6;11(8):1068. doi: 10.3390/antibiotics11081068 (PMC9405050; doi:10.3390/antibiotics11081068)
Supplement: Supplementary file 1 [file antibiotics-11-01068-s001.zip › Supplemental file figure.pdf]

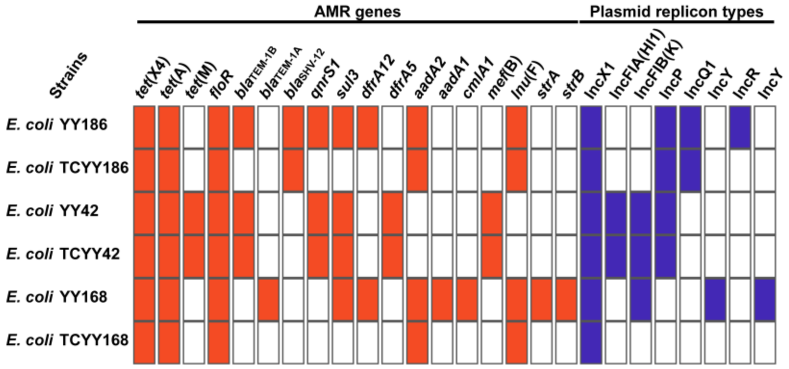

Figure S1. Distribution of antimicrobial-resistance genes and plasmid-replicon types among three *E. coli* isolates and their transconjugants.

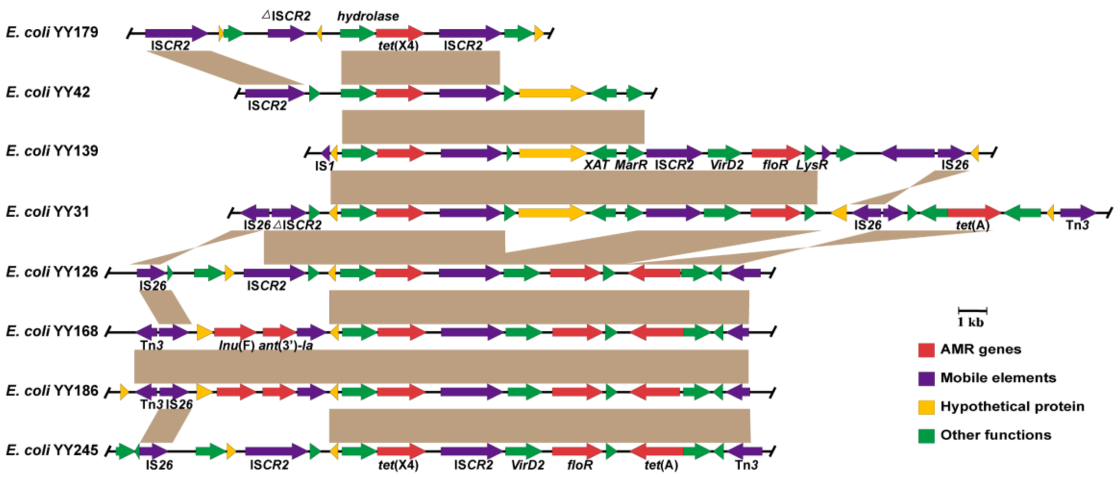

Figure S2. Characterization of *tet*(X4)-bearing genetic environments in eight isolates. Arrows indicate the positions and directions of the genes. Regions with >99% homology are shaded in brown. The *Δ* symbol indicates a truncated gene.

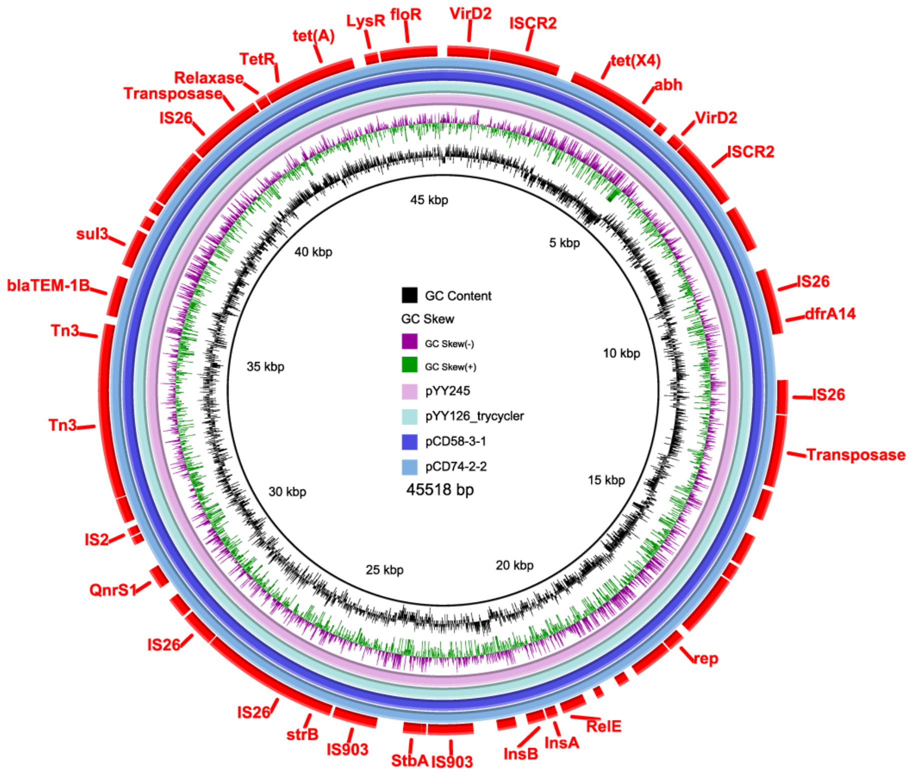

Figure S3 Circular comparison of *tet*(X4)-bearing IncX1 plasmid, pYY245.

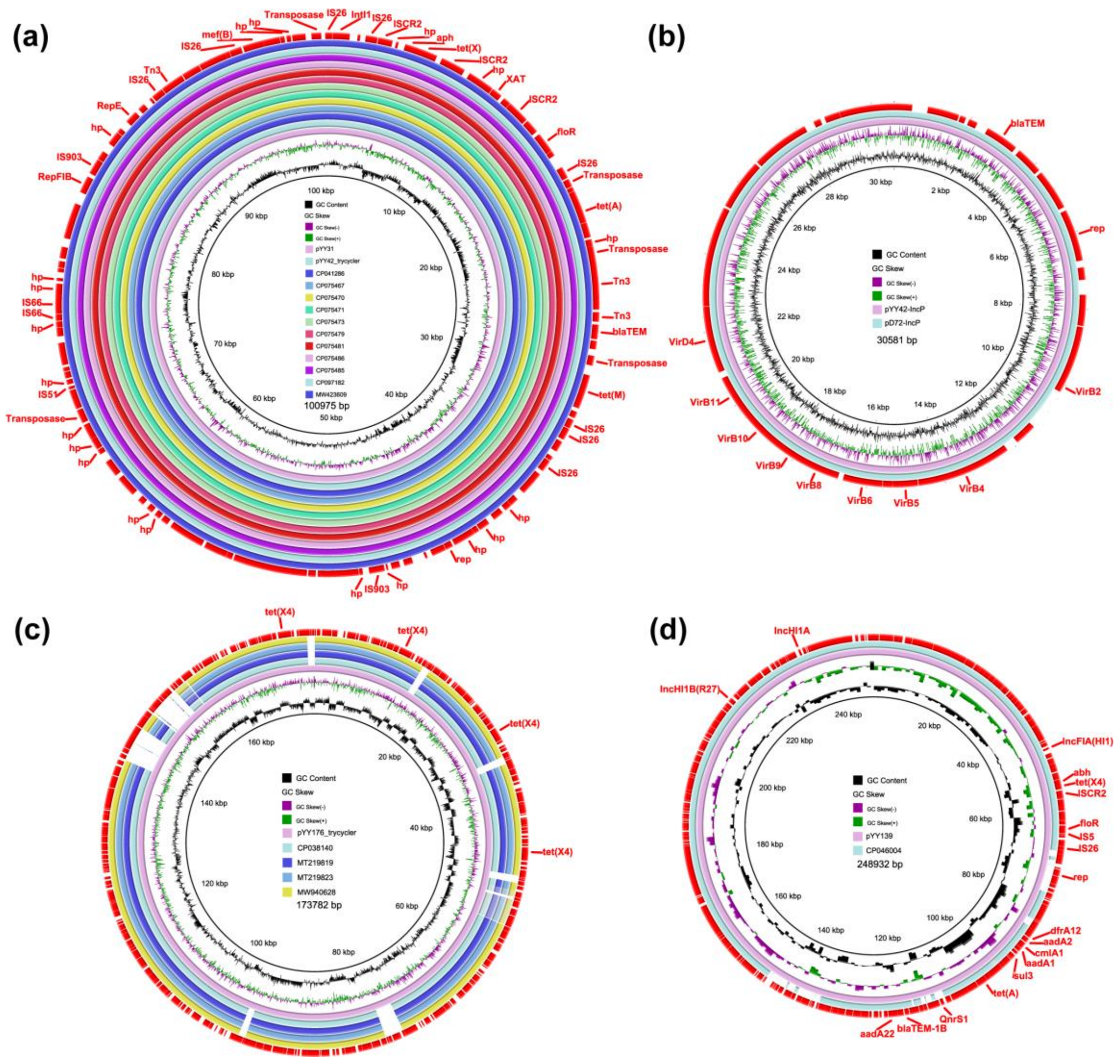

Figure S4 Circular comparison of four plasmids. (a) pYY31, (b) pYY42-IncP, (c) pYY176_trycycler (c), and (d) pYY139.
